# Management of incidental STIC lesions with and without BRCA mutations: a survey of current clinical practice in Austria, Germany, and Switzerland

**DOI:** 10.1007/s00404-026-08442-y

**Published:** 2026-04-20

**Authors:** Natalia Anna Kaufmann, Peter Oppelt, Christoph Grimm, Julia Lastinger, Teresa Eichinger, Philip Trautner, Anja Hartl, Stefan Raidl, Dariga Ramazanova, Caroline Ines Preuss

**Affiliations:** 1 Department of Gynaecology, Obstetrics and Gyn. Endocrinology, Johannes Kepler University Linz, Kepler University Hospital, Altenberger Strasse 69, 4040 Linz and Krankenhausstrasse 26-30, 4020 Linz, Austria; 2 Division of General Gynaecology and Gynaecologic Oncology, Gynaecologic Cancer Unit, Comprehensive Cancer Center, Medical University of Vienna, Spitalgasse 23, 1090 Vienna, Austria; 3 Clinical Institute of Pathology and Molecular Pathology, Johannes Kepler University Linz, Kepler University Hospital, Altenberger Strasse 69, 4040 Linz and Krankenhausstrasse 9, 4020 Linz, Austria; 4 Center for Clinical Studies (CCS Linz) at the Center for Clinical Research, Johannes Kepler University Linz, Krankenhausstrasse 5, 4020 Linz, Austria

**Keywords:** Ovarian cancer, Carcinogenesis, BRCA, HBOC

## Abstract

**Objective:**

The aim of this study was to assess current clinical practices, management, and follow-up care for women with an isolated serous tubal intraepithelial carcinoma (STIC) diagnosis in German-speaking countries.

**Methods:**

An online survey targeting all German-speaking gynecological centers was developed. The survey included single- and multiple-choice questions on hospital data, such as the number of cases per year and certification, as well as detailed questions on two scenarios: a *BRCA1*-positive patient undergoing prophylactic bilateral salpingo-oophorectomy and a patient with an incidental STIC finding after hysterectomy and bilateral salpingectomy.

**Results:**

This survey was answered by 77 physicians. For a patient with a known *BRCA1* mutation and STIC, 89.29% of respondents would perform further diagnostics. The most frequent diagnostic steps would be a CA-125 test (83.64%) and a CT abdomen (63.64%). Further surgery would be performed by 77.78% of respondents, including 75.93% without and 5.56% with lymph node staging. 79.25% would prefer laparoscopic surgery. The majority (90.57%) would not recommend adjuvant therapy. In a patient without a known mutation and STIC, 88.46% of respondents would recommend further examinations. 58.82% would carry out genetic panel testing. Another subsequent surgery would be performed by 76.47%, with 66.67% planning to perform surgery without lymph node staging. 53.06% of respondents would follow up patients for five years. Percentages are reported based on the number of valid responses for each item.

**Conclusion:**

This survey demonstrates differences in the clinical management of isolated STIC across German-speaking countries, highlighting discrepancies between guideline recommendations and real-world practices.

**This study is registered in the German Clinical Trials Register under:**

DRKS00033112

**Supplementary Information:**

The online version contains supplementary material available at 10.1007/s00404-026-08442-y.

## What does this study add to the clinical work

**Table:** 

This survey demonstrates differences in the clinical management of isolated STIC across German-speaking countries, highlighting discrepancies between guideline recommendations and real-world practices, especially regarding surgery, genetic testing, and follow-up care. The findings underline the need for standardized clinical algorithms and consensus-based guidelines to ensure consistent and evidence-informed care for patients with isolated STIC.	

## Introduction

Serous Tubal Intraepithelial Carcinoma (STIC) is a lesion confined to the fallopian tube epithelium characterized by distinct histologic features, such as significant atypia and an aberrant pattern of p53 staining. Since the finding that precursor lesions in the fallopian tube account for a relevant number of high-grade serous ovarian cancer cases, the topic of STIC has become more and more relevant [1, 2]. A key prerequisite for STIC is the establishment of the SEE-FIM protocol. SEE-FIM stands for “Sectioning and Extensively Examining the Fimbriated End” and means that surgical specimens of fallopian tubes are processed using a detailed protocol for embedding and sectioning [3].

The prevalence of STIC seems to be significantly influenced by the presence of a *BRCA* mutation. In previous studies, the prevalence of isolated STIC in *BRCA*-positive patients has been reported to range between 0.6% and 7%. It also occurs in 46% of individuals with spontaneous ovarian cancer and in up to 80% of *BRCA*-positive patients who are diagnosed with high-grade serous ovarian cancer [4–7]. There is currently no reliable information on the prevalence of isolated STIC in women without *BRCA* mutations. Previous studies reported a prevalence of less than 0.01%, and a recent registry analysis from one German federal state likewise documented only a few isolated cases within a two-year period [8].

The importance of STIC is underlined by a recent finding: According to the data from Steenbeek et al., STIC is associated with a high risk of developing peritoneal carcinomatosis (cumulative risk after 5 years: 10.5% versus 0.3%) [9].

Due to the relatively new recognition of STIC and the different prevalence rates between women with and without *BRCA* mutations, current clinical guidelines differ in some aspects or provide only limited recommendations. For example: The German language S3 guideline ‘Diagnosis, Therapy and Aftercare of Malignant Ovarian Tumors’ is limited to the possibility of a staging procedure to exclude high-grade lesions and to face the risk of an already existing invasive process. This is because STIC lesions are not invasive, but they can be the first sign of high-grade serous carcinomas [10]. No explicit recommendation is made regarding lymphadenectomy. In contrast, the European Society of Gynecological Oncology–European Society for Medical Oncology (ESGO-ESMO) consensus clearly advises peritoneal staging without lymph node dissection [11].

Due to the increasing number of genetic counseling and testing, the number of risk-reducing salpingo-oophorectomies is increasing. Additionally, awareness of hereditary breast and ovarian cancer (HBOC) is improving. Thus, STICs are being diagnosed much more frequently. This survey aimed to assess the clinical practice and expertise regarding isolated STIC and determine the current diagnostic and treatment strategy in German-speaking gynecological departments.

## Methods

The questionnaire was developed by a multidisciplinary team of gynecologists, oncologists, and pathologists using SoSci Survey, an open-source platform for non-profit research, and sent to the gynecologic departments via a link to www.soscisurvey.de. A similar survey was conducted in Germany by van der Ven and Linz et al. [12]. This previously published questionnaire provides a structured assessment of diagnostic and therapeutic management of isolated STIC. However, because we additionally aimed to address management according to mutation status, genetic testing, hormone replacement therapy, and follow-up care, we developed a modified questionnaire rather than using the previously published instrument unchanged. The ethics committee was consulted; a formal approval was not required. The project was conducted between January and December 2024 in collaboration with the working group for gynaecological oncology of the German Society for gynaecological oncology, the Swiss Go Trial Group, and WAAGO, as part of the working group for gynaecological oncology in Austria. Each association explicitly referred to the survey in its newsletters. In addition, the link was sent via email to all available German-speaking gynecological centers in Austria and Germany. The list of German-speaking gynecological centers was compiled by internet research. Two reminders were sent. Only German-speaking departments were contacted in Switzerland. Participation was voluntary. To reduce the likelihood of duplicate responses, the survey platform applied standard technical measures. However, repeated participation cannot be completely excluded.

This survey consisted of single- and multiple-choice questions and included eleven general questions about the hospital and its tumor-related data, such as the number of gynecological cancer cases per year and, regarding pathology, the use of SEE-FIM protocol. Subsequently, 16 questions were asked for each of two clinical cases. The first case described a patient with a known *BRCA1* mutation who had undergone risk-reducing salpingo-oophorectomy. The second case described a patient who underwent a hysterectomy including bilateral salpingectomy and was diagnosed with STIC as an incidental finding. The questions covered the main topics of diagnosis, surgical treatment and follow-up. The analysis was carried out by the statisticians of the Competence Center for Clinical Studies at the Johannes Kepler University. The statistical software R (version 4.3.1) was used for the statistical analysis. All analyzed variables were nominal. Hence, for all variables, absolute frequencies and percent of the number of valid observations (= percent without missing values) are reported. The certification of the center was dichotomized (none or at least one) and visualized using stacked bar charts. Because participation in individual survey questions was optional, item-level missing data occurred. Percentages are therefore calculated using the number of valid responses for each item. The number of valid responses is reported alongside percentages in the table to ensure transparency. No statistical tests were performed in this exploratory study. The focus is rather on the qualitative interpretation of the collected data and the classification of the results in the context of expertise. The survey is exploratory in nature and is intended primarily to show trends and tendencies. Because the survey link was distributed via newsletters and mailing lists, and could be forwarded within institutions, the exact number of physicians who received the invitation could not be determined. Consequently, a formal response rate could not be calculated.

## Results

A total of 77 physicians participated in the survey. Of these, 55.56% were from Austria, 27.78% from Germany, and 16.67% from Switzerland. The most common certifications were ‘Onkozert’ (44.16%), German Cancer Society (18.18%), ESGO (10.39%), ‘DOC Zert’ (9.09%), Swiss Cancer League (2.60%) and Austrian Certification Commission (6.49%). 26.09% of the institutions are classified as Care Level I or Care Level II. 18.84% are categorized as Care Level III, while 27.54% are university hospitals.

Regarding clinical guidelines, respondents were asked both about detailed familiarity (“knowledge”) and about whether the guideline is applied within their institutional clinical routine (“use”). Detailed familiarity with the German S3 guideline was reported by 13.56% of respondents, while 86.44% indicated that the guideline is applied in their clinical routine. The ESGO guideline was reported as known by 19.15% and used by 80.85%. The NCCN guideline showed the highest familiarity (51.85%) but was reported to be applied in practice by 48.15% of respondents. In this context, “knowledge” refers to self-reported detailed familiarity with the guideline, whereas “application” denotes its implementation as part of the institutional clinical routine and therefore, it is possible that reported application rates are higher than knowledge rates. Of the respondents, 53.25% stated that they use the SEE-FIM protocol, while 1.30% stated that they do not use it. 28.57% do not know whether this protocol is used in their institution. Opportunistic salpingectomies as part of other procedures are offered by 98.44% of respondents.

The information provided on the first case was as follows: *Patient A, *with a *known BRCA1 mutation and no previous diseases. A prophylactic salpingo-oophorectomy was performed on both sides. In the pathology findings, an STIC was diagnosed in the right tube with otherwise unremarkable findings*. In this case further diagnostic steps would be performed by 89.29% of respondents. As further diagnostic steps, 63.16% would analyze tumor markers and recommend imaging. 71.93% of the respondents would recommend gynecological examination and sonography. When asked which tumor markers should be examined, the answers were as follows: 83.64% CA-125, 23.64% CEA, 12.73% CA19.9, 3.64% CA15.3, 30.91% HE4. Imaging was recommended by 63.16% of the respondents. A subsequent surgery was recommended by 77.78%, whereas 18.52% strictly refused any additional surgery. The following surgical steps were recommended: only 5.56% would recommend systematic lymph node staging during subsequent surgery. In terms of hysterectomy, 24.53% of respondents would generally recommend it if STIC was diagnosed. Recommendations for hysterectomy depending on other factors, like age or mutations, are shown in Table 1. Most respondents (90.57%) do not recommend administering adjuvant systemic therapy.

**Table 1:** Comparison of responses between the two clinical cases. This table shows relative frequencies (percent) and absolute numbers (x/n, valid cases without NA) for selected diagnostic and surgical measures to ensure transparency. Case 1 describes a patient with a known *BRCA1* mutation who underwent risk-reducing salpingo-oophorectomy and was diagnosed with isolated STIC. Case 2 describes a patient with no known genetic risk who underwent hysterectomy with bilateral salpingectomy for benign indications, and STIC was found incidentally

	Case 1	Case 2
Patient background	Patient A, *BRCA1* + , no previous diseases Prophylactic salpingo-oophorectomy (bilateral) STIC in right tube	Patient B, no family history, no previous diseases Hysterectomy + bilateral salpingectomy Leiomyomas + STIC in right tube
Further diagnostics recommended	89.29% (50/56)	88.46% (46/52)
Tumor marker analysis	63.16% (36/57)	71.15% (37/52)
Tumor marker most recommended (CA-125)	83.64% (46/55)	84.31% (43/51)
Imaging recommended	63.16% (36/57)	63.46% (33/52)
Gynecological exam + sonography	71.93% (41/57)	76.92% (40/52)
CT abdomen/pelvis	63.64% (35/55)	70.59% (36/51)
MRI	12.73% (7/55)	15.69% (8/51)
Transvaginal ultrasound	58.18% (32/55)	50.98% (26/51)
CT Thorax	21.82% (12/55)	23.53% (12/51)
PET-CT	5.45% (3/55)	3.92% (2/51)
Bone scintigraphy	0% (0/55)	0% (0/51)
Mammography + ultrasound	36.36% (20/55)	58.82% (30/51)
Mammography + ultrasound + MRI	47.27% (26/55)	15.69% (8/51)
Subsequent surgery recommended	77.78% (42/54)	76.47% (39/51)
Surgical approach: laparoscopy laparotomy robotic	79.25% 5.66% 1.89%	76.47% 7.84% 1.96%
Lymph node staging during surgery	5.56% (3/54)	5.88% (3/51)
Hysterectomy: Generally for serous tubal intraepithelial carcinoma for serous tubal intraepithelial carcinoma and *BRCA* mutation for serous tubal intraepithelial carcinoma and Lynch syndrome for serous tubal intraepithelial carcinoma and completed childbearing for serous tubal intraepithelial carcinoma and age > 40 for serous tubal intraepithelial carcinoma and age > 50	24.53% 9.43% 26.42% 32.08% 1.89% 1.89%	22% 12% 26% 36% 10% 6%
Diagnostic hysteroscopy + curettage instead of hysterectomy	9.43% (5/53)	8% (4/50)
No adjuvant therapy recommended	90.57% (48/53)	96% (48/50)

The information provided on the second case was as follows: *Patient B, unremarkable family history, no previous illnesses. A hysterectomy with bilateral salpingectomy was performed in the case of a completed desire to have children and *a *uterine myoma. The pathology findings included leiomyomas of the uterus and an STIC in the right tube.*

In this case, 88.46% of respondents would recommend further diagnostic steps, 71.15% would analyze tumor markers, and 63.46% would recommend imaging. 76.92% of respondents would perform a gynecological examination including sonography. When asked which tumor markers should be analyzed, the answers were as follows: 84.31% CA-125, 25.49% CEA, 13.73% CA19.9, 7.84% CA15.3, and 27.45% HE4. Imaging was recommended by 63.46%. In this case, the patient’s genetic background was unknown, and the family history was unremarkable. Despite this, 17.65% of respondents would initiate selective testing for *BRCA1* and *BRCA2*, while 58.82% would prefer an extended panel. 23.53% of participants stated that they would only initiate genetic testing in the case of a positive family history. Regarding the role of genetic findings in clinical decisions, 5.88% of respondents stated that they would only perform surgical staging if a pathogenic *BRCA1* or *BRCA2* mutation was present. 25.49% would perform surgical staging if any pathogenic mutation was detected. For 68.63% of the participants, the genetic finding does not play a relevant role in the decision-making process.

Subsequent surgery was recommended by 76.47% of respondents. 5.88% would perform the surgery with lymph node staging. Regarding oophorectomy in a 35-year-old patient with STIC, 37.25% would remove only the affected ovary, while 15.69% would perform bilateral oophorectomy. In case of a proven pathogenic *BRCA* mutation, 39.22% of respondents would remove both ovaries; if another pathogenic mutation was present, 52.94% would seek bilateral removal. In a comparable 45-year-old patient, 58.82% would perform bilateral oophorectomy, 29.41% would do so with a proven *BRCA* mutation, and 58.82% would if another pathogenic mutation was present. Recommendations for hysterectomy depending on other factors are shown in Table 1. Similar to case 1, most respondents (96.0%) did not recommend adjuvant therapy.

**Fig. 1:**
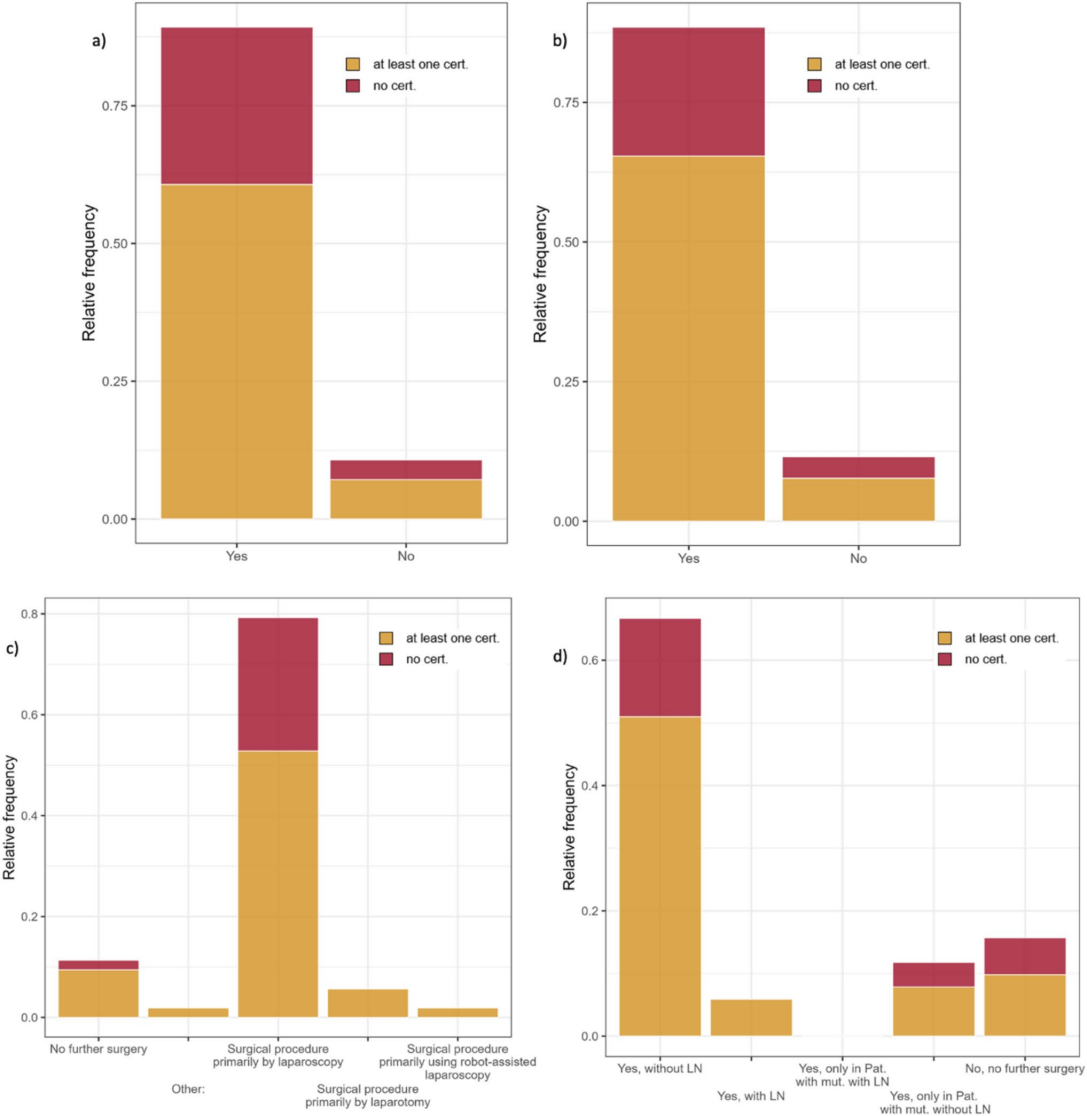
Responses to selected questions by certification status. **a** Patient 1 with known BRCA1 mutation underwent prophylactic bilateral salpingo-oophorectomy. Pathology revealed *STIC* with no other abnormalities. Responses to "Would you recommend further diagnostic work-up?" Color-coded by hospital certification: 89.29% Yes, 10.71% No. **b** Patient 2 with no personal or family history underwent hysterectomy with bilateral salpingectomy for uterine fibroids. Pathology revealed *STIC* along with uterine leiomyomas. Responses to "Would you recommend further diagnostic work-up?" Color-coded by hospital certification: 88.46% Yes, 11.54% No. **c** Patient 1. Responses to: "If you opted for surgery due to *STIC* with a pathogenic BRCA1 mutation, what is your primary surgical approach?" Color-coded by hospital certification: Laparoscopy 79.25, Robot-assisted 1.89%, Laparotomy 5.66%, No further surgery 11.31%, Other (laparoscopy as off-label use) 1.89%. **d** Patient 2. Responses to: "If you opted for surgery due to *STIC*, do you recommend peritoneal staging?" Color-coded by hospital certification: Yes, without lymph node staging 66.67%, with lymph node staging 5.88%, only for patients with pathogenic mutation (without lymph node staging) 11.76%, only for patients with pathogenic mutation (with lymph node staging) 0%, no further surgery 15.69%. *LN* = lymph node staging mut. = pathogenic mutation

The questions on follow-up care related to the general procedure after a diagnosis of STIC, independently of the case scenarios. 40.82% of respondents would perform a gynaecological check-up including transvaginal ultrasonography every three months. 26.53% would recommend check-ups every six months, while 14.29% would prefer annual follow-up. Regular tumor marker tests would be ordered by 24.49% of the participants. Diagnostic laparoscopy as part of follow-up would be performed by 14.29%. 6.12% of respondents stated that they do not perform routine follow-up for serous tubal intraepithelial carcinomas. 10.20% would recommend combined oral contraceptives to premenopausal patients. 26.53% stated that they would arrange follow-up care analogous to that for patients with ovarian cancer. Regarding imaging diagnostics in follow-up care, most respondents (91.84%) see no need for radiological diagnostics as part of follow-up care. When asked how long to follow up patients, 53.06% of respondents chose the 5-year option. 30.61% chose lifelong follow-up. The descriptive comparison between certified and non-certified centers did not reveal clear differences in selected management decisions. The corresponding results are shown in Fig. 1. As the analysis was descriptive and based on a limited sample size, the lack of observed differences between certified and non-certified centers should not be interpreted as evidence of equivalence but rather as hypothesis-generating findings.

## Discussion

The survey’s findings demonstrate the variety of clinical management options for STIC and the present substantial uncertainty on this topic with respect to management and treatment recommendations. According to our survey, most clinicians would perform additional diagnostic work-up and consider further surgery in cases of isolated STIC. A majority would perform peritoneal staging without lymphadenectomy and prefer a minimally invasive surgical approach. In follow-up care, most respondents rely on regular clinical examinations and sonography.

Systematic imaging follow-up after STIC was rejected by most respondents, which reflects the current data situation, as there is no evidence to date for an advantage of intensive imaging surveillance [13]. The relevance of genetic testing is evaluated inconsistently: Although some respondents believe that genetic testing is crucial, almost 70% of respondents do not believe that it is an important factor in planning therapy strategies. This conclusion contrasts with current guidelines, which increasingly take genetic results into account when making decisions about prophylactic measures and follow-up care. In our survey, the majority (58.82%) opted for genetic panel testing. It is crucial to remember that not only patients with *BRCA* mutations are at risk; if panel testing is available, it should be advised for all STIC patients [13–15]. In addition, in 2023, the German Cancer Society included STIC in its checklist for identifying a potential hereditary cancer predisposition for breast or ovarian cancer. According to the checklist, patients with isolated serous tubal intraepithelial carcinomas should undergo genetic testing [16].

In 2020, a similar survey was conducted in Germany by van der Ven and Linz et al. [12]. When compared with this survey, our findings confirm that the management of STIC remains highly inconsistent across clinical practice. Both studies show that clinicians rarely recommend adjuvant chemotherapy and generally favor minimally invasive staging while avoiding systematic lymph node dissection. However, our multinational data indicate a shift toward more extensive diagnostic evaluation, particularly the more frequent use of cross-sectional imaging. More importantly, our survey expands the perspective beyond procedural management by demonstrating that genetic testing plays a substantial but still inconsistently applied role in clinical practice. This aspect reflects the growing relevance of hereditary cancer risk assessment in modern oncologic care. Regarding surgical approaches for serous tubal intraepithelial carcinoma, it was shown in our survey that many respondents only support a hysterectomy in specific circumstances, e.g., when the patient has a positive family history. Decisions for ovarian surgery also differ based on the patient’s age. In general, the majority would perform minimally invasive surgery on the patients, as it offers an opportunity to reduce the morbidity of the operation. The approach is in line with the new recommendations for serous tubal intraepithelial carcinomas management presented at the 2025 ESGO Congress in Rome. Conversely the current S3 guideline explicitly recommends laparotomy as the surgical approach of choice in this clinical context. However, our survey results clearly demonstrate that this recommendation is not being implemented in routine clinical practice. This discrepancy highlights a gap between guideline recommendations and real-world clinical behavior—particularly striking given that 66.23% of respondents reported that they adhere to the S3 guideline. As shown in our survey, current practice reflects the ESGO recommendations more than other guidelines. More than half of the respondents would follow these patients for up to 5 years. Of note, the overall risk of peritoneal carcinomatosis following a STIC diagnosis in the presence of a BRCA mutation seems to increase within the first ten years. Thus, long-term follow-up for at least ten years is supported [9, 17].

This is the first international survey to date focusing on the clinical management of serous tubal intraepithelial carcinomas across all German-speaking countries. The survey reflects real-world clinical practice by capturing data from both certified and non-certified gynecologic departments. We are aware of the limitations of this survey. The number of participants is limited, and the distribution between countries is uneven, which restricts the generalizability of the results. As participation was voluntary, physicians with a particular interest or expertise in STIC may have been more likely to respond, introducing a potential selection bias. In addition, the survey link was distributed via newsletters and mailing lists and could be forwarded within institutions; therefore, the total number of recipients could not be reliably determined, and a formal response rate cannot be calculated. Another limitation is the possibility that multiple physicians from the same institution participated in the survey. Because responses were collected anonymously, clustering by institution could not be assessed and was therefore not accounted for in the analysis.

At the ESGO 2025 Congress, particular attention was drawn to the critical role of peritoneal washing. Current NCCN Guidelines recommend performing pelvic washing for cytology during all risk-reducing surgical procedures [18]. We acknowledge that the increasing importance of pelvic peritoneal washing in guiding therapeutic decisions represents a limitation of our survey. Evidence indicates that up to 15% of patients undergoing risk-reducing surgery present with positive cytology, further underscoring the clinical significance of this high-risk population [19].

To overcome the persistent heterogeneity in STIC management, structured collaborative efforts are required. Prospective multicenter registries could provide standardized data on staging procedures, genetic testing, cytology, follow-up strategies, and long-term outcomes. In this context, the STIC Register represents a valuable opportunity to systematically collect real-world data and support evidence-based recommendations [20]. In addition, a consensus statement developed by gynecologic oncology societies within German-speaking countries may further harmonize current practice. Finally, clarification and updating of existing guidelines, particularly the German S3 guideline, appear necessary to reduce ambiguities and better align recommendations with contemporary evidence and real-world clinical decision-making. The issue is recognized by the major gynecological oncology societies and is increasingly being addressed at both national and international congresses. This trend was already noticeable over the course of our survey.

## Conclusions

It is challenging to determine the most effective strategy of treatment for isolated serous tubal intraepithelial carcinomas. Genetic counseling and germline testing for HBOC remain essential components of care in these patients. There are currently no data on the preferred treatment options for those affected. To conclude, it is evident that current clinical practice remains dependent on individual assessments and expertise rather than standardized guidelines.

## Supplementary Information

Below is the link to the electronic supplementary material.

Supplementary file1 (PDF 251 KB)

## Data Availability

The datasets used during this study are available from the corresponding author on reasonable request.

## Abbreviations

ESGO: European Society of Gynaecological Oncology
ESMO: European Society for Medical Oncology
HBOC: Hereditary breast and ovarian cancer
NCCN: National Comprehensive Cancer Network
SEE-FIM protocol: Sectioning and Extensively Examining the Fimbriated End protocol
STIC: Serous tubal intraepithelial carcinoma
