# Supplementary material for: Management of incidental STIC lesions with and without BRCA mutations: a survey of current clinical practice in Austria, Germany, and Switzerland
Source: Arch Gynecol Obstet. 2026 Apr 20;313(1):169. doi: 10.1007/s00404-026-08442-y (PMC13095959; doi:10.1007/s00404-026-08442-y)
Supplement: Supplementary file 1 — Supplementary file1 (PDF 251 KB) [file 404_2026_8442_MOESM1_ESM.pdf]

Questionnaire - STIC Survey

Palasz Natalia Anna, Preuß Caroline Ines

Please answer the questionnaire from the perspective of your department.

1. In which country do you operate?
   - Germany
   - Austria
   - Switzerland
   - Other: *[Free text field]*

2. Is your hospital/clinic certified? *(multiple answers possible)*
   - OnkoZert
   - Doc-Zert
   - ESGO
   - DKG
   - EUSOMA
   - Swiss Cancer League (KLS)
   - Austrian Certification Commission (ÖZK)
   - Other: *[Free text field]*
   - No specific certification available for cancer diseases

   What type of certification do you have? *(multiple answers possible)*:
   - Genital Cancer Center
   - Breast Cancer Center
   - Familial Breast and Ovarian Cancer Center (FBREK)
   - Cooperation partner of a certified FBREK center

3. Which care level does your hospital belong to? (*multiple answers possible)*
   - Basic or standard care hospital (care level I)
   - Specialist hospital (care level II)
   - Maximum care hospital (care level III)
   - University Hospital

4. How many primary cases of ovarian, fallopian tube and primary peritoneal cancer do you have in your center each year?
   - 1 - 10
   - 11 - 20

- 21 - 30
- 31 - 40
- > 40

5. Which of the recommendations for STIC treatment listed below do you know or use? *(Please check the boxes for know and use separately)*

know	***use***
○ S3 Guideline Ovarian Cancer AWMF	○ S3 Guideline Ovarian Cancer AWMF
○ ESGO - ESMO	○ ESGO - ESMO
○ National Comprehensive Cancer Network	○ National Comprehensive Cancer Network
○ Other: [Free text]	○ Other: [Free text]
○ None of the above	○ None of the above

6. Does your pathology institute use the SEE-FIM protocol (Sectioning and Extensively Examining the FIMbria) for the workup of the tubes?
   - Yes
   - No
   - Unknown
7. Does your hospital offer patients opportunistic salpingectomy as part of other surgical procedures?
   - Yes
   - No
   - Unknown

8. Does your hospital offer prophylactic salpingoophorectomies to patients at high risk of familial breast and ovarian cancer?
   - Yes
   - Yes, with pathogenic BRCA1/2 *mutation*
   - Yes, for pathogenic *BRCA1/2 mutations* and also for other pathogenic mutations e.g. *BRIP1, RAD51C, RAD51D, PALB2, or HNPCC*
   - No
   - Unknown

9. Do you recommend **postoperative hormone (replacement) therapy** after bilateral prophylactic salpingoophorectomy for a **healthy female patient** with a pathogenic *BRCA1 or* BRCA2 *mutation*? *[Multiple answers possible]*
   - Yes, without restriction
   - Yes, only in individual cases
   - Yes, if there is no known family history of breast cancer
   - Yes, with the following restriction: [Free text]
   - No, is not indexed

10. Do you recommend **postoperative hormone (replacement) therapy** to patients after bilateral **salpingoophorectomy for STIC** (provided the patient has no history of breast cancer)? *[Multiple answers possible]*
    - Yes, without restriction
    - Yes, only in individual cases
    - Yes, if there is no known family history of breast cancer
    - Yes, after normal genetic testing
    - Yes, even with a proven pathogenic BRCA1/2 *mutation*
    - Yes, even with a proven pathogenic mutation (other than *BRCA*)
    - Yes, with the following restriction: [Free text]
    - No

11. If a **healthy patient** refuses a bilateral/contralateral prophylactic salpingoophorectomy in a high-risk situation for breast and/or ovarian cancer (e.g. pathogenic mutation, STIC), we recommend the following procedure *[multiple answers possible]*
    - Regular reevaluation by vaginal examination/PAP and transvaginal sonography every 3 months
    - Regular reevaluation by vaginal examination/PAP and transvaginal sonography every 6 months
    - The regular control of tumor markers
    - For STIC: the performance of a diagnostic laparoscopy

      Time interval:
      - 3 months
      - 6 months
      - 12 months
    - Taking an oral contraceptive
    - None of the above options

The following is an age-independent description of a casus.

Case 1) Patient A, known BRCA1 *mutation*, no previous diseases. A prophylactic salpingoophorectomy was performed on both sides. In the pathology findings, an STIC was diagnosed in the right tube with otherwise unremarkable findings.

Diagnostics:

12. Do you carry out further diagnostics when a STIC is diagnosed?
    - Yes
    - No

    If yes, what further diagnostics do you carry out? *[Multiple answers possible]*
    - Tumor markers
    - Imaging
    - Gynecological examination including transvaginal sonography

13. If you take tumor markers when you are diagnosed with STIC, then... *[Multiple answers possible]*
    - CA 125
    - CA 19-9
    - CA 15-3
    - CEA
    - HE 4
    - Tumor markers - other: *[free text]*
    - No, no tumor markers for STIC

14. If you perform another **imaging examination** for the diagnosis of STIC, then... *(multiple answers possible)*
    - CT abdomen/pelvis
    - MRI abdomen/pelvis
    - Transvaginal ultrasound
    - CT thorax
    - PET-CT
    - Bone scintigraphy
    - More: *[Free text]*

15. If not yet performed, do you recommend that the patient undergo breast diagnostics at the time of the initial diagnosis of STIC? *(multiple answers possible)*
    - o Breast sonography only
    - o Mammography only
    - o Breast MRI only
    - o Mammography and breast sonography
    - o Breast sonography and breast MRI
    - o Mammography, breast sonography and breast MRI
    - o No breast diagnostics

Surgical procedure and therapy for STIC:

16. Do you perform further surgery on this patient **with STIC with pathogenic BRCA1/2 mutation** after bilateral salpingoophorectomy?
    - o Yes
    - o No

17. If you have decided to operate on the basis of **STIC,** do you recommend surgical peritoneal staging with multiple PEs from the upper, middle and lower abdomen, omentectomy and irrigation cytology for this patient?
    - o Yes, without lymph node staging
    - o Yes, incl. lymph node staging
    - o No, no further surgery

18. If you have opted for surgery for **STIC with pathogenic *BRCA1/2* mutation**, what is your primary surgical approach for STIC?
    - o Operative procedure primarily by laparoscopy
    - o Surgical procedure primarily using robot-assisted laparoscopy
    - o Surgical procedure primarily by laparotomy
    - o Other: *[Free text]*
    - o No further surgery

19. Since the primary operation was performed **without a hysterectomy**, would you recommend a hysterectomy to the patient? [*Multiple answers possible]*
    - o Yes, generally with STIC
    - o Yes, in patients with a pathogenic *BRCA1/2 mutation*
    - o Yes, for patients with HNPCC
    - o Yes, for patients who have completed their desire to have children

- Yes, for patients aged 40 and over
- Yes, for patients aged 50 and over
- No
- No, I would primarily perform a diagnostic hysteroscopy and fractionated curettage

20. Do you recommend adjuvant systemic therapy for a patient with **STIC with pathogenic BRCA1/2 *mutation***?
    - Yes, carboplatin and paclitaxel
    - Yes, carboplatin monotherapy
    - Yes, other [free text]
    - Yes, only in the context of studies
    - No

The following is an age-independent description of a case, please note the age information for individual questions.

Case 2) Patient B, **unremarkable family history**, no previous illnesses. A hysterectomy with bilateral salpingectomy was performed in the case of a completed desire to have children and uterine myoma. The pathology findings included leiomyomas of the uterus and a STIC in the right tube.

Diagnostics:

21. If this patient is diagnosed with STIC, do you carry out further diagnostics?
    - o Yes
    - o No

    If yes, what further diagnostics do you carry out? *[Multiple answers possible]*
    - o Tumor markers
    - o Imaging
    - o Gynecological examination including transvaginal sonography

22. Do you carry out genetic testing for familial breast and ovarian cancer (germline testing in the blood) regardless of the patient's age?
    - o Yes, only *BRCA1/BRCA2*
    - o Yes, panel testing (multigene analysis e.g. *BRCA1/2, BRIP1, RAD51D, PALB2, HNPCC etc.*)
    - o Yes, if there is a positive family history of breast and/or ovarian cancer
    - o No, not with STIC.

23. If you take tumor markers when you are diagnosed with STIC, then*... [Multiple answers possible]*
    - o CA 125
    - o CA 19-9
    - o CA 15-3
    - o CEA
    - o HE 4
    - o Tumor markers - other: *[free text]*
    - o No, no tumor markers for STIC

24. If you perform another **imaging examination** for the diagnosis of STIC, then... *(multiple answers possible)*
    - CT abdomen/pelvis
    - MRI abdomen/pelvis
    - Transvaginal ultrasound
    - CT thorax
    - PET-CT
    - Bone scintigraphy
    - More: *[Free text]*

25. Do you recommend that this patient undergo breast diagnostics at the time of initial diagnosis of STIC? *(multiple answers possible)*
    - Breast sonography only
    - Mammography only
    - Breast MRI only
    - Mammography and breast sonography
    - Breast sonography and breast MRI
    - Mammography, breast sonography and breast MRI
    - No breast diagnostics

Surgical procedure and therapy for STIC:

26. Do you perform further surgery on a patient **with STIC without a pathogenic BRCA1/2 mutation** after salpingectomy?
    - No
    - Yes

27. Does the result of genetic testing in the presence of STIC play a role for you in the recommendation for further surgical staging?
    - Yes, further surgical staging only for pathogenic *BRCA1/2* mutation
    - Yes, further surgical staging for pathogenic *BRCA1/2 mutation* and also for other pathogenic mutations e.g. *BRIP1, RAD51C, RAD51D, PALB2, or HNPCC*
    - No, it doesn't matter

28. If you have decided to operate on the basis of **STIC,** do you recommend surgical peritoneal staging with multiple PEs from the upper, middle and lower abdomen, omentectomy and irrigation cytology for this patient?
    - Yes, without lymph node staging

- Yes, incl. lymph node staging
- Yes, only in patients with pathogenic mutation without lymph node staging
- Yes, only in patients with pathogenic mutation incl. lymph node staging
- No, no further surgery

29. If you have opted for surgery for **STIC without a pathogenic BRCA1/2 mutation**, what is your primary surgical procedure for STIC?
    - Operative procedure primarily by laparoscopy
    - Surgical procedure primarily using robot-assisted laparoscopy
    - Surgical procedure primarily by laparotomy
    - Other: *[Free text]*
    - No further surgery

30. Would you recommend an ovariectomy to a **35-year-old female patient** with the same case? *[Multiple answers possible]*
    - Yes, only the affected side (right)
    - Yes, on both sides
    - Yes, bilateral in patients with pathogenic *BRCA1/2 mutation*
    - Yes, bilateral in case of proven high-risk situation (pathogenic mutation) for familial breast and ovarian cancer [*BRCA and others*]
    - No

31. Would you recommend an ovariectomy for a **45-year-old patient** with the same case?
    - Yes, only the affected side (right)
    - Yes, on both sides
    - Yes, bilateral in patients with pathogenic *BRCA1/2 mutation*
    - Yes, bilateral in case of proven high-risk situation (pathogenic mutation) for familial breast and/or ovarian cancer *[BRCA and others]*
    - No

32. If only a salpingectomy **without hysterectomy** had been performed in the primary operation in this case study, would you recommend a hysterectomy for this patient? [*Multiple answers possible]*
    - Yes, generally with STIC
    - Yes, in patients with a pathogenic *BRCA1/2 mutation*
    - Yes, for patients with HNPCC
    - Yes, for patients who have completed their desire to have children
    - Yes, for patients aged 40 and over
    - Yes, for patients aged 50 and over

- No
- No, I would primarily perform a diagnostic hysteroscopy and fractionated curettage

33. Do you recommend adjuvant systemic therapy for patients **with STIC without a pathogenic BRCA1/2 mutation**?
    - Yes, carboplatin and paclitaxel
    - Yes, carboplatin monotherapy
    - Yes, other [free text]
    - Yes, only in the context of studies
    - No

Follow-up care for STIC *(case-independent)**:***

34. If you have decided not to perform any further surgery on one of the patients or the other findings are unremarkable, how do you monitor the patients as part of the follow-up care at STIC? *[Multiple answers possible]*
    - Gynecological examination incl. ultrasound every 3 months
    - Gynecological examination incl. ultrasound every 6 months
    - Gynecological examination incl. ultrasound every 12 months
    - the regular control of tumor markers
    - the performance of a diagnostic laparoscopy

      Time interval:
        - 3 months
        - 6 months
        - 12 months
    - No routine follow-up care for STIC
    - We offer the use of an oral contraceptive as an individualized procedure for premenopausal patients
    - Follow-up care as for ovarian cancer
    - Other: *[Free text]*

35. Over what period of time do you provide aftercare for STIC?
    - 1 year
    - 3 years
    - 5 years
    - For life
    - No aftercare for STIC

36. Do you recommend further imaging procedures for a patient with STIC as part of follow-up care? *[Multiple answers possible]*

**Imaging procedure**	**Interval**
- CT	○ Every 3 months
	○ Every 6 months
	○ Every 12 months
- MRI	○ Every 3 months
	○ Every 6 months
	○ Every 12 months
- PET-CT	○ Every 3 months
	○ Every 6 months
	○ Every 12 months
- No radiological diagnostics in the routine	

37. The current breast diagnostics of a patient with a recent initial diagnosis of **STIC** are unremarkable. What do you recommend to the patient for the future regarding the **breast**? (multiple answers possible)
    - o In the case of STIC and inconspicuous genetic testing: rescreening as part of the regular statutory cancer screening examinations/mammography screenings
    - o In the case of STIC and inconspicuous genetic testing: Intensified early detection with an individual risk of developing breast cancer of ≥ 5% in the next 10 years (individual risk calculation is carried out)
    - o With STIC and inconspicuous genetic testing: Intensified early detection (**no** individual risk calculation is carried out)
    - o In case of STIC and detection of a pathogenic mutation: Intensified early detection
    - o In case of STIC and detection of a pathogenic mutation: option of a prophylactic bilateral mastectomy
    - o All patients with evidence of STIC receive intensified early breast screening
    - o None of the points above apply

38. Other recommendations that you offer as a procedure at STIC*: [Free text]*
